# Tetra-μ-acetato-κ^8^ *O*:*O*′-bis­{[2-methyl­sulfanyl-4-(pyridin-4-yl-κ*N*)pyrimidine]­copper(II)}(*Cu—Cu*)

**DOI:** 10.1107/S1600536811029837

**Published:** 2011-07-30

**Authors:** Hai-Bin Zhu, Wen-Na Yang

**Affiliations:** a School of Chemistry and Chemical Engineering, Southeast University, Nanjing 211189, People’s Republic of China

## Abstract

The binuclear title compound, [Cu_2_(CH_3_CO_2_)_4_(C_10_H_9_N_3_S)_2_], comprises a Cu_2_(CH_3_CO_2_)_4_ paddle-wheel core axially bound by two 2-methyl­sulfanyl-4-(pyridin-4-yl)pyrimidine ligands. The complex mol­ecule has an inversion center lying at the mid-point of the Cu—Cu bond.

## Related literature

For a related structure, see: Li *et al.* (2009 ▶).
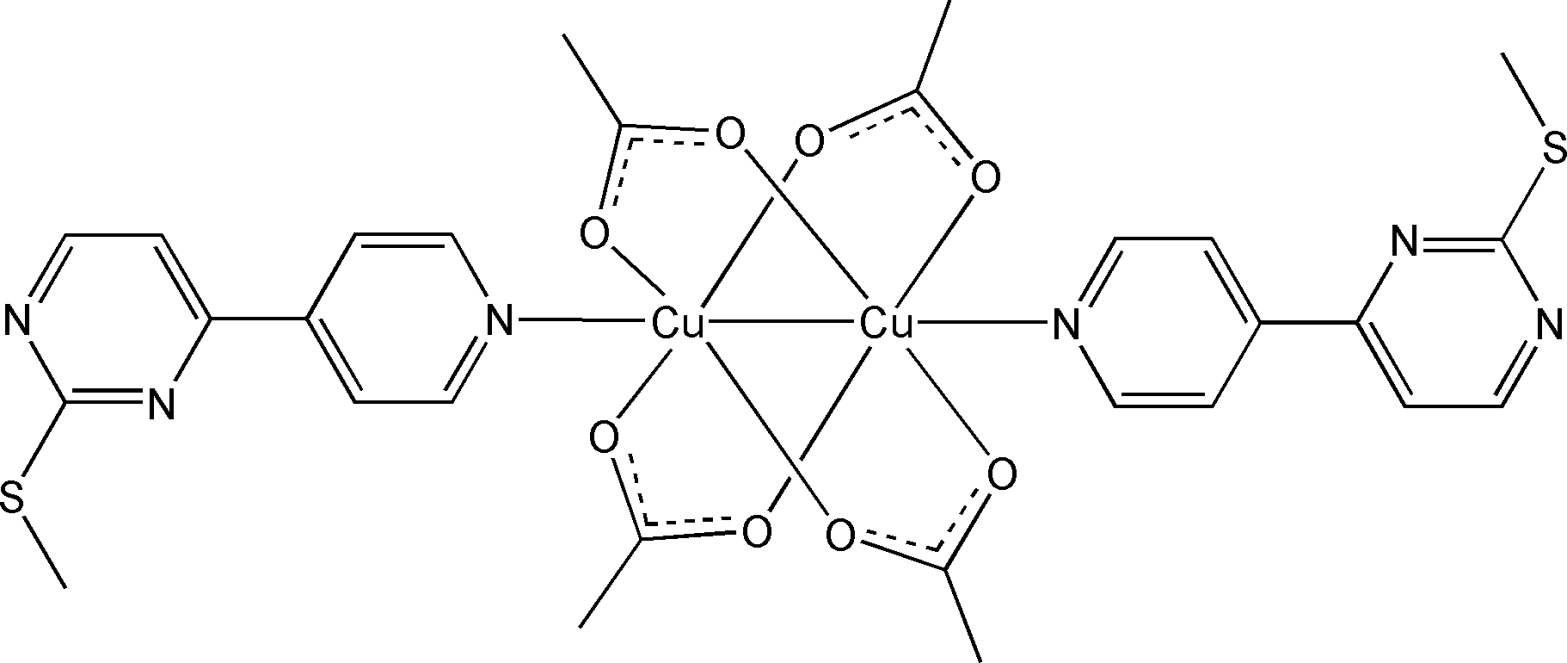


## Experimental

### 

#### Crystal data

[Cu_2_(C_2_H_3_O_2_)_4_(C_10_H_9_N_3_S)_2_]
*M* *_r_* = 769.82
Monoclinic, 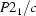

*a* = 15.192 (2) Å
*b* = 13.003 (2) Å
*c* = 8.6108 (13) Å
β = 103.781 (2)°
*V* = 1652.0 (4) Å^3^
*Z* = 2
Mo *K*α radiation
μ = 1.47 mm^−1^
*T* = 298 K
0.41 × 0.25 × 0.18 mm

#### Data collection

Bruker APEXII CCD diffractometer
Absorption correction: multi-scan (*SADABS*; Bruker, 2001 ▶) *T*_min_ = 0.650, *T*_max_ = 0.768
9568 measured reflections
2827 independent reflections
2354 reflections with *I* > 2σ(*I*)
*R*_int_ = 0.024

#### Refinement

*R*[*F* ^2^ > 2σ(*F* ^2^)] = 0.031
*wR*(*F* ^2^) = 0.097
*S* = 1.04
2827 reflections
208 parameters
H-atom parameters constrained
Δρ_max_ = 0.38 e Å^−3^
Δρ_min_ = −0.32 e Å^−3^

### 

Data collection: *APEX2* (Bruker, 2007 ▶); cell refinement: *SAINT-Plus* (Bruker, 2007 ▶); data reduction: *SAINT-Plus*; program(s) used to solve structure: *SHELXS97* (Sheldrick, 2008 ▶); program(s) used to refine structure: *SHELXL97* (Sheldrick, 2008 ▶); molecular graphics: *SHELXTL* (Sheldrick, 2008 ▶); software used to prepare material for publication: *SHELXTL*.

## Supplementary Material

Crystal structure: contains datablock(s) I, global. DOI: 10.1107/S1600536811029837/hy2450sup1.cif

Structure factors: contains datablock(s) I. DOI: 10.1107/S1600536811029837/hy2450Isup2.hkl

Additional supplementary materials:  crystallographic information; 3D view; checkCIF report

## Figures and Tables

**Table 1:** Selected bond lengths (Å)

Cu1—O1^i^	1.970 (2)
Cu1—O2	1.967 (2)
Cu1—O3^i^	1.974 (2)
Cu1—O4	1.967 (2)
Cu1—N1	2.183 (2)
Cu1—Cu1^i^	2.6299 (7)

Symmetry code: (i) 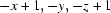.

**Fig. 1.**
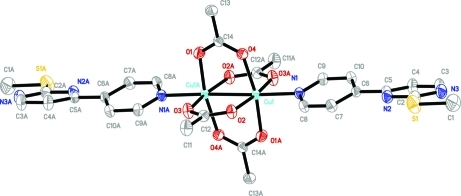
The molecular structure of the title compound, with displacement ellipsoids drawn at the 30% probability level. H atoms have been omitted for clarity. [Symmetry code: (A) 1-x, -y, 1-z.]

## supplementary crystallographic information

### Comment

In our previous work, we have reported a mononuclear Cu(II) complex with 4-(pyridin-4-yl)pyrimidine-2-sulfonate ligand (Li *et al.*, 2009). Herein, we report a binuclear Cu(II) coordination compound with 2-(methylthio)-4-(pyridin-4-yl)pyrimidine (*L*) ligand. The title compound lies on an inversion center with a Cu—Cu separation of 2.6299 (7) Å (Fig. 1). The *L* ligand acts as a terminal ligand *via* its pyridine N atom, with a Cu—N distance of 2.183 (2) Å (Table 1).

### Experimental

A mixture of Cu(CH_3_CO_2_)_2_ (0.1 mmol), *L* (0.1 mmol) in ethanol (15 ml) was stirred for 20 min at room temperature. After filtration, the mother liquid was allowed to stand for one week to give blue crystals suitable for X-ray diffraction analysis.

### Refinement

H atoms bound to C atoms were positioned geometrically and refined as riding atoms, with C—H = 0.93 (aromatic) and 0.96 (methyl) Å and *U*_iso_(H) = 1.2(1.5 for methyl)*U*_eq_(C).

### Crystal data

**Table:**

[Cu_2_(C_2_H_3_O_2_)_4_(C_10_H_9_N_3_S)_2_]	*F*(000) = 788
*M**_r_* = 769.82	*D*_x_ = 1.548 Mg m^−^^3^
Monoclinic, *P*2_1_/*c*	Mo *K*α radiation, λ = 0.71073 Å
Hall symbol: -P 2ybc	Cell parameters from 2827 reflections
*a* = 15.192 (2) Å	θ = 2.3–25.5°
*b* = 13.003 (2) Å	µ = 1.47 mm^−^^1^
*c* = 8.6108 (13) Å	*T* = 298 K
β = 103.781 (2)°	Block, blue
*V* = 1652.0 (4) Å^3^	0.41 × 0.25 × 0.18 mm
*Z* = 2	

### Data collection

**Table:**

Bruker APEXII CCD diffractometer	2827 independent reflections
Radiation source: fine-focus sealed tube	2354 reflections with *I* > 2σ(*I*)
graphite	*R*_int_ = 0.024
φ and ω scans	θ_max_ = 25.0°, θ_min_ = 2.1°
Absorption correction: multi-scan (*SADABS*; Bruker, 2001)	*h* = −15→18
*T*_min_ = 0.650, *T*_max_ = 0.768	*k* = −15→15
9568 measured reflections	*l* = −10→10

### Refinement

**Table:**

Refinement on *F*^2^	Primary atom site location: structure-invariant direct methods
Least-squares matrix: full	Secondary atom site location: difference Fourier map
*R*[*F*^2^ > 2σ(*F*^2^)] = 0.031	Hydrogen site location: inferred from neighbouring sites
*wR*(*F*^2^) = 0.097	H-atom parameters constrained
*S* = 1.04	*w* = 1/[σ^2^(*F*_o_^2^) + (0.0614*P*)^2^ + 0.3763*P*] where *P* = (*F*_o_^2^ + 2*F*_c_^2^)/3
2827 reflections	(Δ/σ)_max_ < 0.001
208 parameters	Δρ_max_ = 0.38 e Å^−^^3^
0 restraints	Δρ_min_ = −0.32 e Å^−^^3^

### Fractional atomic coordinates and isotropic or equivalent isotropic displacement parameters (Å^2^)

**Table:**

	*x*	*y*	*z*	*U*_iso_*/*U*_eq_	
Cu1	0.42790 (2)	0.03013 (2)	0.39231 (4)	0.03313 (14)	
O2	0.39177 (14)	−0.11545 (15)	0.3826 (3)	0.0474 (5)	
O4	0.50560 (15)	−0.00064 (17)	0.2452 (2)	0.0462 (5)	
N1	0.31254 (16)	0.08776 (18)	0.2112 (3)	0.0380 (5)	
O1	0.62838 (15)	−0.05037 (17)	0.4261 (3)	0.0522 (6)	
O3	0.51441 (14)	−0.16651 (15)	0.5641 (3)	0.0518 (6)	
C14	0.5845 (2)	−0.03341 (19)	0.2841 (4)	0.0396 (7)	
C8	0.2390 (2)	0.0311 (2)	0.1478 (4)	0.0440 (7)	
H8	0.2376	−0.0368	0.1810	0.053*	
C6	0.16582 (19)	0.1693 (2)	−0.0149 (3)	0.0377 (6)	
C10	0.2425 (2)	0.2277 (2)	0.0483 (3)	0.0427 (7)	
H10	0.2459	0.2956	0.0164	0.051*	
C9	0.3132 (2)	0.1843 (2)	0.1582 (3)	0.0410 (7)	
H9	0.3644	0.2243	0.1980	0.049*	
C12	0.4403 (2)	−0.1827 (2)	0.4653 (4)	0.0412 (7)	
C7	0.1662 (2)	0.0681 (2)	0.0368 (4)	0.0477 (7)	
H7	0.1169	0.0256	−0.0042	0.057*	
N2	0.02885 (17)	0.1470 (2)	−0.2225 (3)	0.0491 (6)	
C5	0.08668 (19)	0.2131 (2)	−0.1331 (3)	0.0417 (7)	
C4	0.0726 (2)	0.3178 (3)	−0.1478 (4)	0.0573 (9)	
H4	0.1123	0.3640	−0.0846	0.069*	
S1	−0.11441 (7)	0.09520 (9)	−0.43675 (14)	0.0806 (3)	
N3	−0.0610 (2)	0.2871 (3)	−0.3527 (4)	0.0620 (8)	
C13	0.6315 (3)	−0.0562 (3)	0.1536 (4)	0.0579 (9)	
H13A	0.5910	−0.0426	0.0517	0.087*	
H13B	0.6494	−0.1271	0.1590	0.087*	
H13C	0.6842	−0.0133	0.1664	0.087*	
C2	−0.0421 (2)	0.1878 (3)	−0.3268 (4)	0.0514 (8)	
C11	0.4090 (2)	−0.2928 (2)	0.4448 (5)	0.0609 (9)	
H11A	0.4514	−0.3358	0.5166	0.091*	
H11B	0.4050	−0.3141	0.3366	0.091*	
H11C	0.3504	−0.2986	0.4681	0.091*	
C3	−0.0023 (3)	0.3513 (3)	−0.2593 (5)	0.0657 (10)	
H3	−0.0127	0.4216	−0.2707	0.079*	
C1	−0.2020 (2)	0.1704 (4)	−0.5637 (5)	0.0900 (14)	
H1A	−0.2452	0.1254	−0.6301	0.135*	
H1B	−0.1757	0.2145	−0.6298	0.135*	
H1C	−0.2319	0.2114	−0.4990	0.135*	

### Atomic displacement parameters (Å^2^)

**Table:**

	*U*^11^	*U*^22^	*U*^33^	*U*^12^	*U*^13^	*U*^23^
Cu1	0.0360 (2)	0.0322 (2)	0.0284 (2)	0.00451 (13)	0.00199 (15)	−0.00098 (13)
O2	0.0452 (12)	0.0375 (11)	0.0524 (13)	−0.0012 (9)	−0.0028 (10)	0.0009 (9)
O4	0.0511 (13)	0.0567 (12)	0.0316 (11)	0.0104 (10)	0.0111 (10)	0.0027 (9)
N1	0.0412 (14)	0.0382 (13)	0.0322 (13)	0.0043 (10)	0.0039 (11)	0.0004 (10)
O1	0.0528 (13)	0.0680 (15)	0.0362 (12)	0.0198 (11)	0.0116 (11)	0.0003 (10)
O3	0.0536 (14)	0.0350 (10)	0.0552 (13)	−0.0022 (10)	−0.0099 (11)	−0.0011 (9)
C14	0.0538 (19)	0.0307 (14)	0.0372 (17)	0.0042 (13)	0.0166 (15)	0.0001 (12)
C8	0.0453 (18)	0.0382 (15)	0.0444 (18)	0.0003 (13)	0.0024 (15)	0.0087 (13)
C6	0.0379 (16)	0.0397 (15)	0.0339 (15)	0.0062 (12)	0.0057 (13)	0.0037 (12)
C10	0.0484 (18)	0.0342 (14)	0.0410 (17)	0.0016 (13)	0.0019 (14)	0.0030 (12)
C9	0.0419 (16)	0.0369 (15)	0.0393 (16)	0.0005 (12)	0.0001 (13)	−0.0026 (12)
C12	0.0482 (18)	0.0357 (15)	0.0396 (17)	−0.0016 (13)	0.0103 (14)	−0.0075 (13)
C7	0.0403 (17)	0.0469 (17)	0.0503 (19)	−0.0071 (14)	−0.0002 (15)	0.0053 (14)
N2	0.0401 (15)	0.0544 (15)	0.0480 (16)	0.0030 (12)	0.0009 (13)	0.0101 (12)
C5	0.0365 (16)	0.0485 (16)	0.0384 (17)	0.0052 (13)	0.0056 (14)	0.0069 (13)
C4	0.053 (2)	0.0488 (18)	0.062 (2)	0.0087 (16)	−0.0018 (17)	0.0036 (16)
S1	0.0607 (6)	0.0805 (7)	0.0812 (8)	−0.0150 (5)	−0.0214 (5)	0.0158 (6)
N3	0.0477 (17)	0.0690 (19)	0.0622 (19)	0.0113 (14)	−0.0007 (15)	0.0157 (15)
C13	0.077 (3)	0.057 (2)	0.047 (2)	0.0175 (18)	0.0314 (19)	0.0038 (16)
C2	0.0376 (17)	0.067 (2)	0.0468 (19)	0.0017 (15)	0.0045 (15)	0.0123 (16)
C11	0.063 (2)	0.0362 (16)	0.076 (2)	−0.0064 (15)	0.0006 (19)	−0.0080 (16)
C3	0.062 (2)	0.052 (2)	0.076 (3)	0.0151 (18)	0.001 (2)	0.0134 (19)
C1	0.045 (2)	0.126 (4)	0.082 (3)	−0.004 (2)	−0.020 (2)	0.018 (3)

### Geometric parameters (Å, °)

**Table:**

Cu1—O1^i^	1.970 (2)	C12—C11	1.505 (4)
Cu1—O2	1.967 (2)	C7—H7	0.9300
Cu1—O3^i^	1.974 (2)	N2—C2	1.338 (4)
Cu1—O4	1.967 (2)	N2—C5	1.335 (4)
Cu1—N1	2.183 (2)	C5—C4	1.379 (4)
Cu1—Cu1^i^	2.6299 (7)	C4—C3	1.373 (5)
O2—C12	1.251 (4)	C4—H4	0.9300
O4—C14	1.241 (4)	S1—C2	1.748 (4)
N1—C9	1.337 (4)	S1—C1	1.796 (4)
N1—C8	1.341 (4)	N3—C2	1.330 (5)
O1—C14	1.265 (4)	N3—C3	1.341 (5)
O3—C12	1.257 (4)	C13—H13A	0.9600
C14—C13	1.498 (4)	C13—H13B	0.9600
C8—C7	1.366 (4)	C13—H13C	0.9600
C8—H8	0.9300	C11—H11A	0.9600
C6—C10	1.388 (4)	C11—H11B	0.9600
C6—C7	1.389 (4)	C11—H11C	0.9600
C6—C5	1.491 (4)	C3—H3	0.9300
C10—C9	1.372 (4)	C1—H1A	0.9600
C10—H10	0.9300	C1—H1B	0.9600
C9—H9	0.9300	C1—H1C	0.9600
			
O4—Cu1—O2	88.92 (9)	O2—C12—C11	118.1 (3)
O4—Cu1—O1^i^	168.10 (9)	O3—C12—C11	116.5 (3)
O2—Cu1—O1^i^	89.58 (9)	C8—C7—C6	119.9 (3)
O4—Cu1—O3^i^	89.87 (9)	C8—C7—H7	120.0
O2—Cu1—O3^i^	168.30 (8)	C6—C7—H7	120.0
O1^i^—Cu1—O3^i^	89.20 (10)	C2—N2—C5	116.5 (3)
O4—Cu1—N1	96.46 (9)	N2—C5—C4	121.1 (3)
O2—Cu1—N1	97.57 (9)	N2—C5—C6	117.4 (3)
O1^i^—Cu1—N1	95.44 (9)	C4—C5—C6	121.5 (3)
O3^i^—Cu1—N1	94.13 (9)	C3—C4—C5	117.5 (3)
O4—Cu1—Cu1^i^	82.34 (7)	C3—C4—H4	121.3
O2—Cu1—Cu1^i^	85.43 (6)	C5—C4—H4	121.3
O1^i^—Cu1—Cu1^i^	85.77 (7)	C2—S1—C1	103.5 (2)
O3^i^—Cu1—Cu1^i^	82.88 (6)	C2—N3—C3	114.7 (3)
N1—Cu1—Cu1^i^	176.77 (7)	C14—C13—H13A	109.5
C12—O2—Cu1	121.89 (19)	C14—C13—H13B	109.5
C14—O4—Cu1	125.8 (2)	H13A—C13—H13B	109.5
C9—N1—C8	116.8 (2)	C14—C13—H13C	109.5
C9—N1—Cu1	119.7 (2)	H13A—C13—H13C	109.5
C8—N1—Cu1	123.46 (19)	H13B—C13—H13C	109.5
C14—O1—Cu1	65.15 (17)	N3—C2—N2	127.2 (3)
C12—O3—Cu1	66.66 (15)	N3—C2—S1	119.7 (3)
O4—C14—O1	125.0 (3)	N2—C2—S1	113.1 (3)
O4—C14—C13	117.9 (3)	C12—C11—H11A	109.5
O1—C14—C13	117.1 (3)	C12—C11—H11B	109.5
N1—C8—C7	123.3 (3)	H11A—C11—H11B	109.5
N1—C8—H8	118.4	C12—C11—H11C	109.5
C7—C8—H8	118.4	H11A—C11—H11C	109.5
C10—C6—C7	117.0 (3)	H11B—C11—H11C	109.5
C10—C6—C5	121.6 (3)	N3—C3—C4	123.0 (3)
C7—C6—C5	121.4 (3)	N3—C3—H3	118.5
C9—C10—C6	119.5 (3)	C4—C3—H3	118.5
C9—C10—H10	120.3	S1—C1—H1A	109.5
C6—C10—H10	120.3	S1—C1—H1B	109.5
N1—C9—C10	123.5 (3)	H1A—C1—H1B	109.5
N1—C9—H9	118.2	S1—C1—H1C	109.5
C10—C9—H9	118.2	H1A—C1—H1C	109.5
O2—C12—O3	125.4 (3)	H1B—C1—H1C	109.5
			
O4—Cu1—O2—C12	81.3 (2)	Cu1—O4—C14—C13	177.6 (2)
O1^i^—Cu1—O2—C12	−86.9 (2)	C9—N1—C8—C7	−1.4 (4)
O3^i^—Cu1—O2—C12	−2.9 (6)	Cu1—N1—C8—C7	178.6 (2)
N1—Cu1—O2—C12	177.7 (2)	C7—C6—C10—C9	−0.9 (4)
Cu1^i^—Cu1—O2—C12	−1.1 (2)	C5—C6—C10—C9	179.2 (3)
O2—Cu1—O4—C14	−85.3 (2)	C8—N1—C9—C10	2.1 (4)
O1^i^—Cu1—O4—C14	−2.5 (6)	Cu1—N1—C9—C10	−178.0 (2)
O3^i^—Cu1—O4—C14	83.0 (2)	C6—C10—C9—N1	−0.9 (4)
N1—Cu1—O4—C14	177.2 (2)	Cu1—O2—C12—C11	−177.5 (2)
Cu1^i^—Cu1—O4—C14	0.2 (2)	N1—C8—C7—C6	−0.4 (5)
O4—Cu1—N1—C9	−80.7 (2)	C10—C6—C7—C8	1.5 (4)
O2—Cu1—N1—C9	−170.5 (2)	C5—C6—C7—C8	−178.6 (3)
O1^i^—Cu1—N1—C9	99.2 (2)	C2—N2—C5—C4	0.6 (4)
O3^i^—Cu1—N1—C9	9.6 (2)	C2—N2—C5—C6	179.4 (3)
O4—Cu1—N1—C8	99.3 (2)	C10—C6—C5—N2	160.4 (3)
O2—Cu1—N1—C8	9.5 (2)	C7—C6—C5—N2	−19.5 (4)
O1^i^—Cu1—N1—C8	−80.8 (2)	C10—C6—C5—C4	−20.7 (4)
O3^i^—Cu1—N1—C8	−170.4 (2)	C7—C6—C5—C4	159.4 (3)
O4—Cu1—O1^i^—C14^i^	4.2 (6)	N2—C5—C4—C3	−0.9 (5)
O2—Cu1—O1^i^—C14^i^	86.9 (2)	C6—C5—C4—C3	−179.7 (3)
O3^i^—Cu1—O1^i^—C14^i^	−81.4 (2)	C3—N3—C2—N2	−1.7 (5)
N1—Cu1—O1^i^—C14^i^	−175.5 (2)	C3—N3—C2—S1	178.5 (3)
Cu1^i^—Cu1—O1^i^—C14^i^	1.5 (2)	C5—N2—C2—N3	0.8 (5)
O4—Cu1—O3^i^—C12^i^	−82.5 (2)	C5—N2—C2—S1	−179.4 (2)
O2—Cu1—O3^i^—C12^i^	1.5 (6)	C1—S1—C2—N3	−0.4 (3)
O1^i^—Cu1—O3^i^—C12^i^	85.6 (2)	C1—S1—C2—N2	179.7 (2)
N1—Cu1—O3^i^—C12^i^	−179.0 (2)	C2—N3—C3—C4	1.2 (5)
Cu1^i^—Cu1—O3^i^—C12^i^	−0.2 (2)	C5—C4—C3—N3	0.0 (6)

Symmetry codes: (i) −*x*+1, −*y*, −*z*+1.
